# INNOVATIVE CARE ENVIRONMENTS FOR PEOPLE WITH DEMENTIA

**DOI:** 10.1093/geroni/igac059.1289

**Published:** 2022-12-20

**Authors:** Hilde Verbeek, Bram de Boer, Marie Boltz

## Abstract

As part of the ongoing culture movement within long-term care several innovative care concepts for people with dementia are developing. These concepts radically change their physical, social and/or organizational environment in order to align care services with needs and demands of people with dementia, and providing meaningful activities. The current symposium will discuss the effects and possible working mechanisms of innovative caring environments for people with dementia across three European countries and the US. The symposium will start with a presentation describing the Homestead care model in the Netherlands, which is a care model that was developed following a co-creation study focused on translating scientific knowledge on nursing home care environments into practice. This is followed by three presentations about the effects of three types of innovative care environments for people with dementia: (1) farm based day care in Norway, (2) shared housing arrangements in Germany, (3) green care farms providing 24-hour nursing care in the Netherlands. The three studies on the effects of innovative care environments present a variety of designs (case study, cluster randomized controlled multi-center intervention study, and a qualitative study with semi-structured interviews) to study the effects on various outcomes (activities, physical effort, social interaction, mood, the number of hospital admissions for people with dementia, quality of life, challenging behavior, risk of falls, stabilization of cognitive abilities, daily life). The symposium will conclude with a reflection on these innovative care concepts from a US perspective.

